# Activation of liver X receptor inhibits the development of pulmonary carcinomas induced by 3-methylcholanthrene and butylated hydroxytoluene in BALB/c mice

**DOI:** 10.1038/srep27295

**Published:** 2016-06-02

**Authors:** Qixue Wang, Lei Sun, Xiaoxiao Yang, Xingzhe Ma, Qi Li, Yuanli Chen, Ying Liu, Di Zhang, Xiaoju Li, Rong Xiang, Yuquan Wei, Jihong Han, Yajun Duan

**Affiliations:** 1 Department of Neurosurgery, The General Hospital of Tianjin Medical University, Tianjin, China; 2 College of Life Sciences, Nankai University, Tianjin, China; 3 College of Biomedical Engineering, Hefei University of Technology, Hefei, China; 4 School of Medicine, Nankai University, Tianjin, China; 5 Collaborative Innovation Center for Biotherapy, Sichuan University, Chengdu, China; 6 The State Key Laboratory of Medicinal Chemical Biology, College of Life Sciences and Collaborative Innovation Center for Biotherapy, Nankai University, Tianjin, China

## Abstract

We previously reported that LXR ligand, T0901317, inhibited the growth of inoculated Lewis lung carcinoma in C57BL/6 mice by activating IFN-γ production. However, the effects of T0901317 on carcinogen-induced pulmonary carcinomas remain unknown. In this study, we initially conducted a statistical analysis on the data of human lung cancer samples extracted from the TCGA database, and determined that survival rate/time of lung cancer patients and grade of lung adenocarcinoma were positively and negatively related to lung IFN-γ levels, respectively. We then determined the inhibitory effects of T0901317 on mouse pulmonary carcinomas induced by 3-methylcholanthrene (MCA) and butylated hydroxytoluene (BHT) or urethane. We found that T0901317 reduced morbidity and mortality in MCA/BHT-injected BALB/c mice by inhibiting lung adenocarcinoma. T0901317 also protected C57BL/6 mice, but not IFN-γ deficient (IFN-γ^−/−^, C57BL/6 background) mice, against MCA/BHT-induced lung hyperplasia/inflammation. In addition, we determined that T0901317 inhibited urethane-induced lung tumors in BABL/c mice. Furthermore, we determined that T0901317 prevented metastasis of 4T1 breast cancer cells in BALB/c mice. Administration of T0901317 substantially increased serum IFN-γ levels and lung IFN-γ expression in BABL/c and C57BL/6 mice. Taken together, our study demonstrates that LXR inhibits MCA/BHT-induced pulmonary carcinomas in BABL/c mice and the inhibition is associated with induction of IFN-γ production.

Interferon-γ (IFN-γ) is a cytokine with multiple biological functions, such as anti-tumorigenesis and immunomodulation^1^. Thus, tumor growth in IFN-γ insensitive mice is faster than wild type mice^2^. STAT (signal transducers and activators of transcription) is a downstream molecule in the IFN-γ pathway, whereas RAG2 (recombination activating gene 2) is necessary for adaptive immunity development. However, the mice with genetic deletion of both RAG2 and STAT expression still display similar tumor incidence to either RAG2 or STAT alone deficient mice, indicating the extensive overlapping anti-tumorigenic effects of both IFN-γ and adaptive immune system^3^. In addition, in the absence of adaptive immunity, IFN-γ secreted by NK cells can promote the formation of M1 macrophages, a cell type which is essential for the innate immunity and cancer immunoediting activity^4^.

Liver X receptor (LXR), a ligand-activated transcription factor, plays an important role in cholesterol and lipid biosynthesis and metabolism. LXR has also been demonstrated to have important functions in immune system^5^,^6^. We previously reported that activation of LXR by ligand, T0901317, induces IFN-γ expression in different cell types *in vitro* and in different tissues *in vivo*. Administration of wild type mice with T0901317 significantly increased serum IFN-γ levels. Moreover, we determined that T0901317 inhibited the growth of inoculated Lewis lung carcinoma (LLC1) tumors in C57BL/6 wild type mice but not IFN-γ deficient (IFN-γ^−/−^, C57BL/6 background) mice, suggesting that the inhibition of tumor growth is associated with induction of IFN-γ production^7^.

Induction of tumorigenesis by 3-methylcholanthrene (MCA) and butylated hydroxytoluene (BHT) is an experimental model that is used to investigate the formation of human adenocarcinoma and the involved mechanisms in animals. In this model, MCA causes K-ras mutation, while BHT can promote inflammation which enhances formation of lung tumors^8^,^9^. The sensitivity to MCA/BHT-induced lung tumors are different among the mouse strains with an order of A/J > BABL/c > C57BL/6^10^,^11^. Interestingly, more MCA/BHT-induced tumors can be found in mice lacking IFN-γ expression than wild type mice which implies that the endogenous IFN-γ production may protect the animals against MCA/BHT-induced tumorigenesis^2^,^3^.

The effects of LXR activation on the development of carcinogen-induced carcinomas are still not clear. The statistical analysis on the data of human lung cancer samples extracted from the TCGA database demonstrates that the survival rate or time of lung cancer patients is correlated with lung IFN-γ expression. In contrast, the grade of lung adenocarcinoma is inversely related to lung IFN-γ expression. Therefore, we anticipated that LXR-activated endogenous IFN-γ production may play an important role to protect animals against carcinogen-induced pulmonary carcinomas. In this study, we used MCA/BHT-induced pulmonary tumors as a model to determine if LXR ligand can inhibit tumorigenesis, and the inhibition is related to activation of IFN-γ production.

## Results

### IFN-γ expression in the lung is inversely correlated to progression of lung cancer

**Figure 1:**
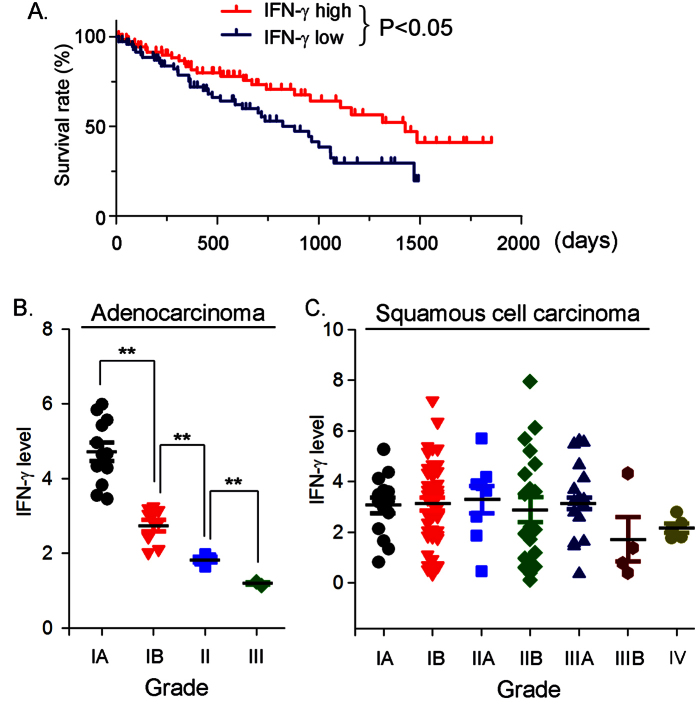
The inverse correlation between the lung IFN-γ expression and the occurrence or grade progression of lung cancer. (**A**) the data of about 150 patient samples with lung squamous cell carcinomas or lung adenocarcinomas was retrieved from the TCGA database and divided into two groups based on lung IFN-γ levels: 50% samples are in the “IFN-γ high” group and another 50% samples are in the “IFN-γ low” group. The survival rate was plotted *vs.* survival time in each group; (**B**) the data from 33 patient samples with lung adenocarcinomas was further divided based on grade of the disease. The IFN-γ level in sub-groups was plotted *vs.* grade of lung adenocarcinoma. **P < 0.01; (**C**) the data from 116 patient samples with lung squamous cell carcinomas was also further divided based on grade of the disease, and the IFN-γ level in sub-groups was plotted *vs.* grade of the lung squamous cell carcinoma. No significant difference (P > 0.05) was determined among the sub-groups.

We previously reported that activation of LXR by T0901317 induced IFN-γ expression both *in vitro* and *in vivo*. Furthermore, we determined that T0901317 administration inhibited growth of inoculated LLC1 tumors in C57BL/6 wild type mice but not mice lacking IFN-γ expression (IFN-γ^−/−^, C57BL/6 background) which indicates that the inhibition of tumor growth by T0901317 is dependent, at least in part, on the activation of IFN-γ production. Although the importance of IFN-γ expression in inhibition of tumor growth has been well investigated in animal models, the relationship between lung IFN-γ expression and progression of human lung cancers is not clear. To explore it, we retrieved the gene expression data of Agilent G4502A_07_3 from the TCGA database (https://tcga-data.nci.nih.gov/tcga/), and obtained data of about 150 patient samples who were clearly diagnosed having either lung squamous cell carcinomas (116 samples) or lung adenocarcinomas (33 samples). Based on IFN-γ levels in the lung of these patients, we divided the data into following two groups (~75 samples/group): 1) “IFN-γ high” group: the lung IFN-γ levels in this group are in top 50% of all samples; 2) “IFN-γ low” group: the lung IFN-γ levels in this group are in bottom 50% of all samples. In each group, we plotted the survival rate (%) *vs.* survival time (days) after diagnosis, and then conducted a statistical analysis between the two groups. The results demonstrate that the survival rate of patients in the “IFN-γ high” group was higher than the survival rate in patients in the “IFN-γ low” group (Fig. 1A, P < 0.05), at the same survival time after diagnosis.

We then divided the data of these patient samples into “group of lung adenocarcinomas” (33 samples) and “group of lung squamous cell carcinomas” (116 samples). The samples in each group were further divided into sub-groups according to grade of the disease. We then plotted the IFN-γ levels in each sub-group *vs.* grade of the disease. The results in Fig. 1B clearly show that there is an inverse correlation between the grade of lung adenocarcinoma and the lung IFN-γ level. In contrast, although there is a moderate decrease of IFN-γ level along with the progression of lung squamous cell carcinoma, the changes are not statistically different (Fig. 1C). Taken together, the above results indicate a possible inverse association between lung IFN-γ expression and development of lung cancer which might be in a lung cancer type-dependent manner.

### LXR activation reduces lung adenocarcinomas in BABL/c mice

**Figure 2:**
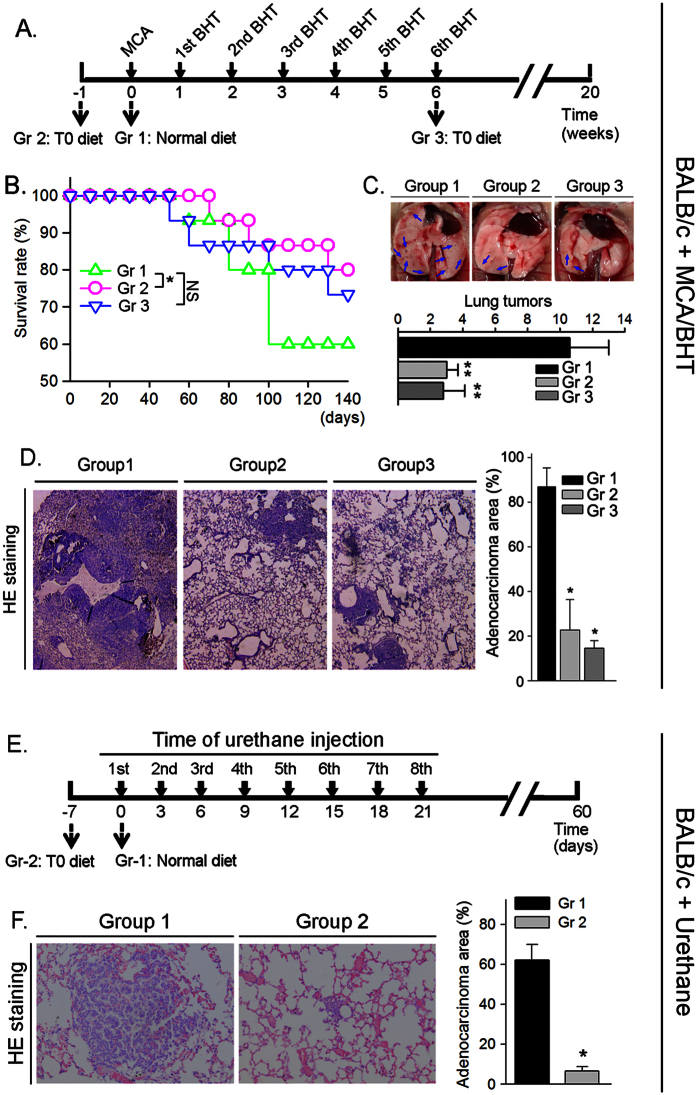
T0901317 inhibits formation of carcinogen-induced lung tumors in BALB/c wild type mice. (**A–D**) BALB/c mice were divided into 3 groups (15 mice/group) and received the scheduled MCA/BHT injections. (**A**) the animals also received the following treatment: mice in Group 1 (Gr 1) were fed normal chow; mice in Group 2 (Gr 2) and Group 3 (Gr 3) were fed normal chow containing T0901317 (5 mpk) one week before and 6 weeks after MCA injection, respectively; (**B**) the death of MCA/BHT-injected mice in each group was daily checked and the survival rate was plotted *vs*. time after MCA injection. *P < 0.05, NS: not significant difference; (**C**) representative photographs of lungs from each group after 20 weeks of MCA injection (top panel). The blue arrows indicate the visible tumors on the surface of the lung. The lung tumors formed in each mouse were counted and the mean of tumor number in each group was plotted (low panel, n = 5; **P < 0.01 *vs.* Group 1); (**D**) lung sections from each group were prepared and conducted HE stained (left panel); the area filled with tumors was determined as % of whole section (right panel). *P < 0.05 *vs.* group 1 (n = 5); (**E**) BALB/c mice were divided into 2 groups (15 mice/group) and fed normal chow or normal chow containing T0901317 (5 mpk). One week later, all the mice were i.p. injected with urethane (1 g/kg bodyweight) once every 3 days for 8 times; (**F**) at the end of the experiment, the urethane-injected mouse lung sections were prepared following by HE staining and determination of tumor area (% of whole section). *P < 0.05 *vs.* Group 1 (n = 15).

The accumulated evidence has demonstrated that activation of LXR may inhibit tumorigenesis. To determine if LXR activation by a ligand can inhibit the development of carcinogen-induced lung adenocarcinomas, we randomly divided BALB/c wild type mice into 3 groups (15 mice/group) and injected the animals with MCA solution. One week later, the animals were injected with BHT solution once a week for consecutive six weeks. Mice also received the following treatment (Fig. 2A): Group 1 (Gr 1), mice were fed normal chow; Group 2 (Gr 2), mice were fed normal chow containing T0901317 at 5 mg/kg bodyweight (mpk) one week before the MCA injection; and Group 3 (Gr 3), mice were started T0901317 (5 mpk) feeding at the same time of the last BHT injection (or 6 weeks after MCA injection). During the treatment, we daily checked dead animal(s). As shown in Fig. 2B, compared to control group (Gr 1), the groups receiving T0901317 treatment (Gr 2 and Gr 3) had increased survival rates, indicating that LXR activation can protect BALB/c mice against the MCA/BHT-induced death.

At the end of experiment (~20 weeks), totally there were 6 mice in Group 1 died. In contrast, 3 and 4 mice were found dead in Group 2 and Group 3 (Fig. 2B and Table 1), respectively. In addition, after histological evaluation on the lung sections prepared from the survived mice at the end of experiment, we found more mice having tumors in Group 1 than Group 2 or Group 3 (Table 1, the microadenomas with size >200 μm^2^ in lung sections were defined as tumors). Among the mice bearing microadenomas, the external appearance clearly displays several visible tumors in the lung surface of MCA/BHT-injected BABL/c mice, while such tumors were substantially reduced by T0901317 treatment in Groups 2 and 3 (Fig. 2C). The histological results in Fig. 2D show disordered alveolar architecture and more than 80% area of the section was occupied by the dense cellular masses and cellular atypia in Group 1 mice bearing tumors. The morphological appearance in Group 1 suggests formation of focal adenocarcinomas in the lung^12^. In contrast, the morphology in most of the areas in the lung sections from the tumor-bearing mice in Group 2 or Group 3 remained normal, and the area occupied by the dense cellular masses and cellular atypia was significantly decreased (Fig. 2D). Thus, the development of MCA/BHT-induced adenocarcinomas was substantially inhibited by T0901317 treatment.

To determine if LXR activation can inhibit pulmonary tumorigenesis in a carcinogen dependent manner, we randomly divided BALB/c mice into 2 groups (15 mice/group) and injected them with another carcinogen, urethane solution (1 g/kg bodyweight)^13^,^14^, once every 3 days for 8 times. Mice in Group 1 were fed normal chow, while the animals in Group 2 were fed normal chow containing T0901317 (5 mpk) and the feeding was started one week before the first urethane injection (Fig. 2E). After two months treatment, we collected lung samples and determined formation of tumors. As shown in Fig. 2F, the results of HE staining demonstrate that adenocarcinoma area in lung section was much bigger in Group 1 mice (>60%) than Group 2 mice (<10%) indicating LXR activation can prevent lung tumorigenesis induced by different carcinogens.

### Inhibition of carcinogen-induced pulmonary carcinomas is associated with activation of IFN-γ production

**Figure 3:**
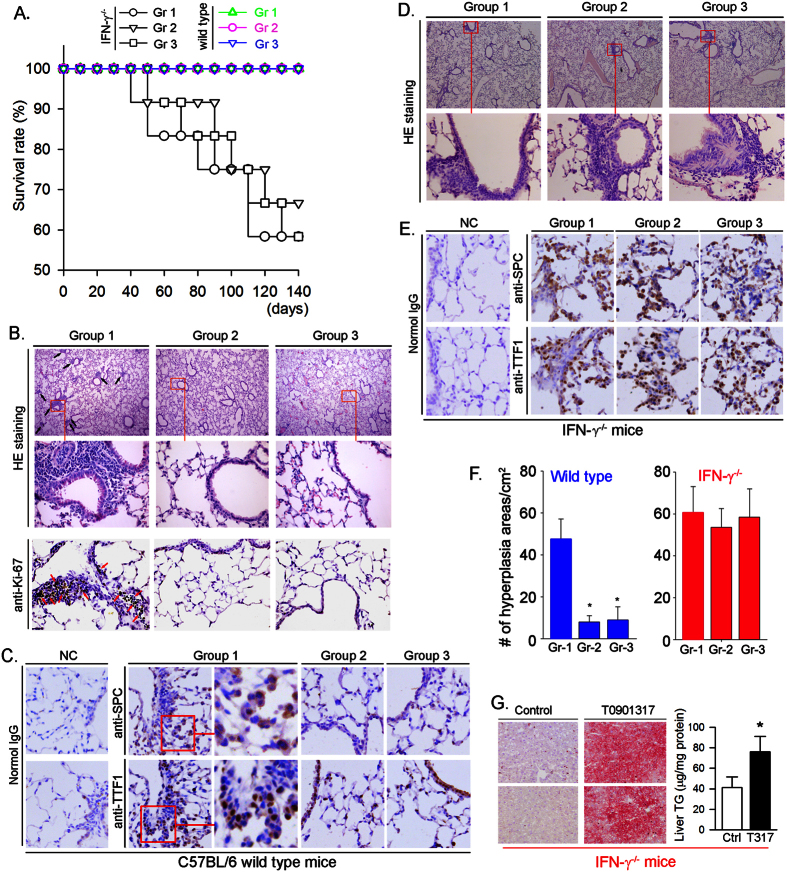
T0901317 inhibits development of atypical hyperplasia and inflammation in C57BL/6 wild type mice, but not IFN-γ^−/−^ mice. C57BL/6 wild type mice or IFN-γ^−/−^ (C57BL/6 background) mice were randomly divided into 3 groups (C57BL/6 wild type mice: 15 mice/group; IFN-γ^−/−^ mice: 12 mice/group) and received the same treatment as indicated in Fig. 2A: Group 1 (Gr 1), fed normal chow; Group 2 (Gr 2), fed normal chow containing T0901317 (5 mpk) from one week before MCA injection; and Group 3 (Gr 3), fed normal chow containing T0901317 (5 mpk) from the 6^th^ BHT (or 6 weeks after MCA) injection. (**A**) the death of mice in either C57BL/6 mice or IFN-γ^−/−^ mice was daily checked after MCA injection. No significant difference was determined among groups either of C57BL/6 mice or IFN-γ^−/−^ mice. At the end of experiment, the lung sections were prepared and conducted HE staining: B (top panel), C57BL/6 mice; (**D**) surviving IFN-γ^−/−^ mice. Expression of Ki-67 ((**B**) low panel), SPC ((**C**) up panel) and TTF1 ((**C**) low panel) in lung sections of C57BL/6 mice were determined by immunohistochemical staining with the corresponding primary antibody. The enlarged images (right panel of Group 1, (**C**)) demonstrate that SPC and TTF1 localize in cellular cytosol and nuclei, respectively; (**E**) expression of SPC and TTF1 in lung sections of IFN-γ^−/−^ mice was determined by immunohistochemical staining; (**F**) areas with hyperplasia/inflammation and size >200 μm^2^ were counted from more than 3 lung sections in each of C57BL/6 wild type mice and IFN-γ^−/−^ mice. *P < 0.05 *vs.* Gr 1; (**G**) IFN-γ^−/−^ mice were divided into two groups (5 mice/group) and fed normal chow (Control, Ctrl) or normal chow containing T0901317 at 5 mpk (T0901317, T317), respectively, for two weeks. Lipid content in the liver was determined by Oil Red O staining (left panel) and TG quantitative assay (right panel), *P < 0.05 (n = 5).

Several mechanisms including activation of IFN-γ production can make contributions to the anti-tumorigenic properties of LXR^7^,^15^,^16^. Because the IFN-γ deficient BALB/c mice are not available, we had to use C57BL/6 wild type mice and IFN-γ deficient (IFN-γ^−/−^, C57BL/6 background) mice to determine the role of IFN-γ production in the inhibition of MCA/BHT-induced pulmonary carcinomas by T0901317 treatment. After the same treatment as BALB/c wild type mice (Fig. 2A), all MCA/BHT-injected C57BL/6 wild type mice fed either normal chow or normal chow containing T0901317 were alive (Fig. 3A, color curves), which is consistent with the nature that C57BL/6 wild type mice are less sensitive than BALB/c wild type mice to the MCA/BHT-induced tumorigenesis. In contrast, deficiency of IFN-γ expression led to more than 40% mice died when they received MCA/BHT injection, suggesting the critical role of endogenous IFN-γ production in protection of animals against tumorigenesis. Furthermore, IFN-γ^−/−^ mice receiving T0901317 treatment either before or after MCA injection still demonstrate similar survival rates to IFN-γ^−/−^ mice without T0901317 treatment (Fig. 3A, black curves) suggesting that T0901317 does not protect IFN-γ^−/−^ mice against MCA/BHT-induced mortality. The results also imply that the protection of BALB/c wild type mice against carcinogen-induced pulmonary tumors (Fig. 2) by T0901317 might be related to induction of IFN-γ production.

The results of histological evaluation on lung sections demonstrate that MCA/BHT treatment resulted in crowded alveolar epithelial cells and alveolar septa surrounded by anisokaryosis and karyomegaly cells in Group 1 C57BL/6 wild type mice (left top and middle panels of Fig. 3B), which indicates the formation of atypical hyperplasia. To confirm it, we determined Ki-67 expression by immunohistochemical staining the lung sections of Group 1 mice, and observed many proliferating cells in the hyperplasia areas (left low panel, Fig. 3B). Furthermore, we confirmed it by determining SPC and TTF1 (two markers for lung tumor) positive cells in the hyperplasia areas (Fig. 3C, middle panel). In addition, the enlarged images (right panel of Group 1, Fig. 3C) show that expression of SPC and TTF1 is in cytoplasm of type II alveolar epithelial cells and nuclei of type II airway epithelial cells, respectively. However, the results in the panel of Group 1 also demonstrate that many cells in the hyperplasia areas are neither SPC nor TTF-1 positive which suggests that BHT induced the infiltration of inflammatory cells. Therefore, MCA/BHT injection leads to formation of a mixture of hyperplasia and inflammation. In contrast, the development of hyperplasia or inflammation induced by MCA/BHT injection was substantially attenuated in the mice receiving T0901317 treatment (Group 2 and Group 3: top middle and right panels of Fig. 3B; right two panels of Fig. 3C). The images in the low panel of Fig. 3C for Group 2 and Group 3 also show the normal TTF1 expression by airway epithelial cells.

Compared to wild type mice, the results in Fig. 3D,E indicate that the development of hyperplasia and inflammation was similar between IFN-γ^−/−^ mice receiving MCA/BHT alone injection (Group 1) and the animals receiving MCA/BHT injection plus T0901317 treatment (Group 2 and Group 3). In addition, the number counting the areas (>200 μm^2^) of hyperplasia/inflammation in lung sections demonstrate that T0901317 substantially reduced it in C57BL/6 wild type mice (left panel, Fig. 3F), but not IFN-γ^−/−^ mice (right panel, Fig. 3F).

Although T0901317 treatment had little effect on high expression of SPC or TTF1 in the lung sections of IFN-γ^−/−^ mice (Fig. 3E), it still induced lipogenesis in the liver of IFN-γ^−/−^ mice (Fig. 3G) which indicates that expression of other LXR target genes than IFN-γ in the animals, such as fatty acid synthase (FASN) and sterol-responsive element binding protein 1c (SREBP1c), was still activated by T0901317 treatment.

**Figure 4:**
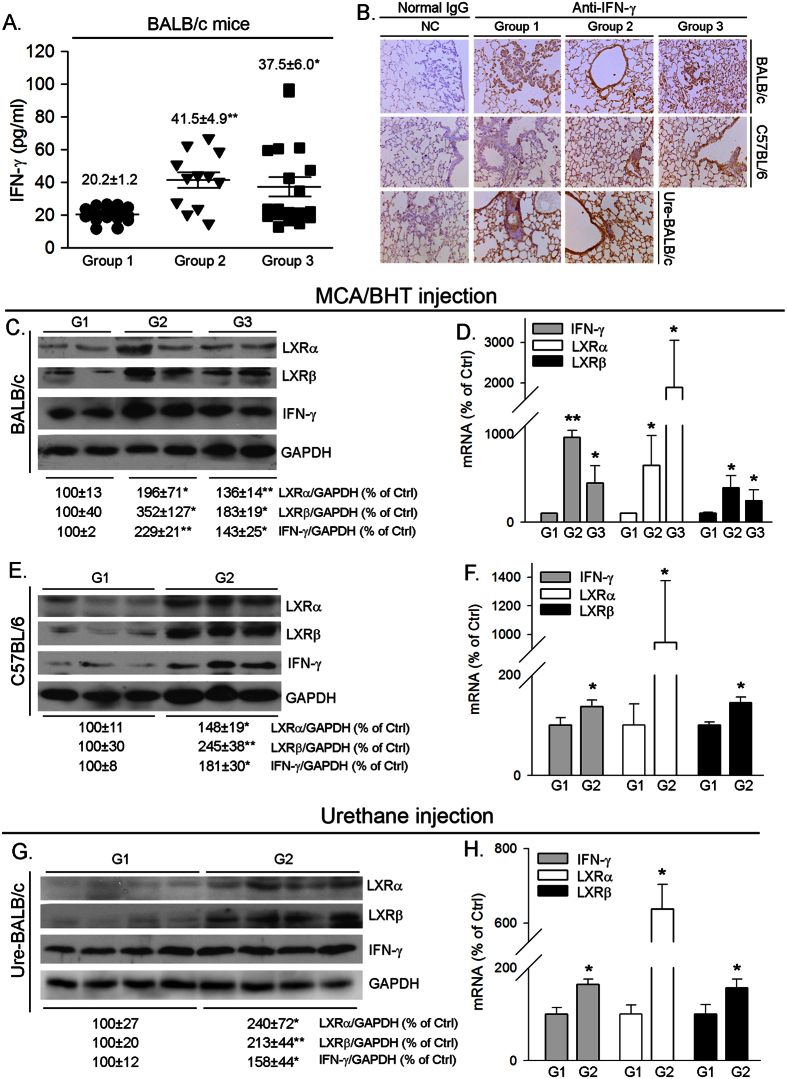
T0901317 increases IFN-γ production in wild type mice. BALB/c mice were i.p. injected with MCA/BHT as indicated in Fig. 2A and urethane as indicated in Fig. 2E, respectively; C57BL/6 wild type mice were i.p. injected with MCA/BHT as indicated in Fig. 2A. All the mice received the following treatment simultaneously: Group 1 (G1), normal chow; Group 2 (G2), normal chow containing T0901317 (5 mpk) one week before MCA/BHT or urethane injection; Group 3 (G3), normal chow containing T0901317 (5 mpk) 6 weeks after MCA injection. Blood and lung samples from BALB/c mice and lung samples from C57BL/6 mice were collected and used to conduct the following assays. (**A**) serum IFN-γ concentrations in BALB/c mice were determined using an ELISA assay kit. *P < 0.05; **P < 0.01 *vs.* Group 1 (n ≥ 20, each sample was determined in duplicate); (**B**) IFN-γ levels in the lung of both BABL/c mice (top panel, MCA/BHT injection; and bottom panel, urethane injection) and C57BL/6 mice (middle panel, MCA/BHT injection) was determined by immunohistochemical staining; (**C,E,G**) expression of IFN-γ, LXRα and LXRβ protein in the lung of BABL/c mice (**C,G**) and C57BL/6 wild type mice (**E**) were determined by Western blot, respectively. The representative images of Western blot were presented. The bands in all Western blots were subject to scanning and density of the band for LXRα, LXRβ or IFN-γ protein was normalized by the density of GAPDH band in the corresponding sample and then statistically analyzed. The expression of LXRα, LXRβ or IFN-γ protein in Group 1 (G1) was defined as 100%, *P < 0.05; **P < 0.01 *vs.* G1 (n = 3); (**D**), (**F,H**) expression of IFN-γ, LXRα and LXRβ mRNA in the lung of BABL/c mice (**D,H**) and C57BL/6 mice (**F**) were determined by real time RT-PCR, *P < 0.05; **P < 0.01 *vs.* G1 (n = 5).

To further link the inhibition of carcinogen induced adenocarcinomas in BABL/c mouse lung or hyperplasia/inflammation in C57BL/6 mouse lung to the activation of IFN-γ production, we determined changes in serum IFN-γ levels and lung IFN-γ expression. Similar to our previous report in C57BL/6 wild type mice^7^, we determined that T0901317 increased serum IFN-γ levels in BALB/c wild type mice (Fig. 4A). Compared to the animals receiving carcinogens alone treatment (Group 1, Fig. 4B), the results of immunohistochmical staining demonstrate that IFN-γ expression in the lung of either MCA/BHT-injected BALB/c wild type mice or C57BL/6 wild type mice, or urethane-injected BALB/c wild type mice, was increased by T0901317 treatment (Group 2 or/and Group 3, Fig. 4B). To semi-quantitatively analyze the effect of T0901317 treatment on lung LXRα, LXRβ and IFN-γ protein and mRNA expression, we prepared total protein and RNA from lung samples of each group. The results of Western blot demonstrate that T0901317 treatment activated expression of LXRα and LXRβ protein. Associated with LXR activation, expression of IFN-γ protein was increased, in both BALB/c mice (Fig. 4C,G) and C57BL/6 mice (Fig. 4E). Similarly, we determined that T0901317 treatment activated expression of lung LXRα, LXRβ and IFN-γ mRNA in both BALB/c mice (Fig. 4D,H) and C57BL/6 mice (Fig. 4F). Taken together, the results in Figs 3 and 4 suggest that inhibition of carcinogen-induced lung adenocarcinomas or hyperplasia/inflammation in wild type mice by T0901317 treatment can be associated with induction of endogenous IFN-γ production.

### Activation of LXR inhibits growth of inoculated tumor cells in cell-type dependent manner

**Figure 5:**
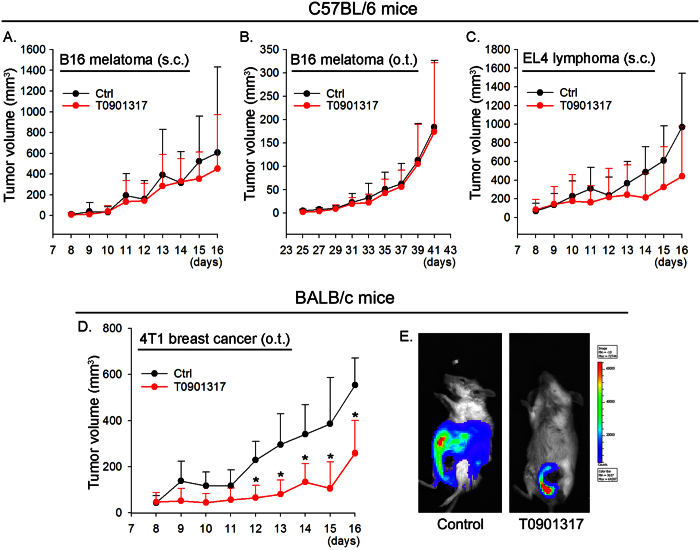
Inhibition of inoculated tumor cells by LXR activation is cell-type dependent. C57BL/6 wild type mice (**A–C**) or BALB/c wild type mice (**D,E**) were pre-fed normal chow (Ctrl, Control) or normal chow containing T0901317 (5 mpk) for one week followed by inoculation of tumor cells at different positions of mouse body. (**A**) B16 cells at the right flank; (**B**) B16 cells at the plantar region of the unilateral hind paws; (**C**) EL4 cells at the right flank; (**D,E**) 4T1 cells at the nipple of female BALB/c mouse. All the animals were daily checked tumor growth. *P < 0.05 *vs.* control (n = 15); (**E**) after 16 days of 4T1 cell injection, mice were i.p. injected with Luciferin solution and then scanned for growth and metastasis of 4T1 tumors. The representative images were presented.

We previously demonstrated that T0901317 inhibited growth of inoculated LLC1 tumors in C57BL/6 wild type mice by activating IFN-γ production^7^. To further determine if T0901317 inhibits growth of inoculated tumors is in tumor cell type or inoculation method dependent manner, C57BL/6 wild type mice received inoculation of B16 cells either by subcutaneous (s.c.) injection at the right flank (Fig. 5A) or orthotopic (o.t.) injection at the plantar region of the unilateral hind paws (Fig. 5B). We determined that T0901317 had little effect on the growth of B16 cells which were either s.c. or o.t. injected. We further determined that T0901317 did not influence the growth of EL4 cells which were s.c. injected at the right flank of mouse (Fig. 5C). In contrast, the growth of o.t. injected 4T1 breast cancer cells at the nipple of female BALB/c mouse was substantially inhibited by T0901317 treatment (Fig. 5D). In addition, at the end of experiment (16 days after injection), we determined that there was a necrotic core around the nipple where 4T1 cells were injected and the proliferating cells distributed in most areas of the main body in control mice (left panel, Fig. 5E) indicating the metastasis occurred. However, T0901317 treatment reduced tumor size and limited the growth within a very local area (right panel, Fig. 5E) suggesting that T0901317 inhibits 4T1 tumor metastasis in BALB/c wild type mice. Taken together, the results in Fig. 5 demonstrate that T0901317 inhibits growth of inoculated tumor cells which might be in cell type, but not inoculation method, dependent manner. In addition, the dose of T0901317 used may play an important role in anti-tumorigenesis since it has been reported that T0901317 can inhibit growth of multiple types of tumors at very higher doses^15^.

## Discussion

Activation of LXR has been demonstrated to play an important role in regulation of cholesterol and lipid synthesis and metabolism. The induction of ATP-binding cassette transporter family members, such as ABCA1 and ABCG1, by LXR activation can enhance reverse cholesterol transport and reduce atherosclerosis. Thus, synthetic LXR ligands are considered as a novel strategy for atherosclerosis treatment. In addition, LXR activity has been implicated in the status of other diseases. The effects of LXR on tumorigenesis have been intensively investigated with controversial results reported. There are two isoforms of LXR, LXRα and LXRβ. Blocking tumor-mediated LXRα signaling can restore dentritic cell (DC) migration to draining lymph nodes, where DCs activate effective antitumor T cells which are responsible for tumor rejection^17^. In contrast, other reports demonstrate that LXR can inhibit various cancers^15^,^16^,^18^. These controversial observations indicate tumor type, animal model and dose of LXR ligand used may influence the effect of LXR on tumorigenesis differently.

T0901317 suppresses melanoma at high doses (20–100 mpk) after a long term treatment^15^. However, treatment of mice with T0901317 at 10 mpk for 4 days can markedly increase hepatic very low-density lipoprotein-triglyceride content, and the prolonged treatment induces a severe fatty liver^19^. We previously determined that T0901317 at 5 mpk significantly increased IFN-γ production and inhibited growth of s.c. injected LLC1 cells^7^. In the present study, we still used T0901317 at 5 mpk to determine its effect on MCA/BHT-induced tumorigenesis in different animal models, which may help to dissect the role of IFN-γ and minimize the undesired effects induced by T0901317 at high doses.

The inhibitory effects of LXR on growth of different tumors might be related to the mechanisms in which LXR functions in a dose-dependent manner. For example, high angiogenic activity can be often detected in tumor progression, and LXR reduces angiogenesis by restraining cholesterol-dependent vascular endothelial growth factor receptor-2 compartmentation and signaling. Therefore, compared to our study demonstrating the inhibition of LLC1 tumor growth by T0901317 at 5 mpk is completed by activating IFN-γ production, the inhibition by T0901317 at 20 mpk is attributed to the anti-angiogenic properties of LXR^20^. Similar to our results in 4T1 tumor model, a previous report shows that LXR significantly reduces proliferation of several other types of human breast cancer cells^21^. The divergence of anti-tumorigenic properties of LXR in the above tumor models might be related to the different roles played by IFN-γ. In 4T1 tumor-bearing mice, IFN-γ producing T cells contribute to increased myeloid derived suppressor cells after cyclophosphamide treatment^22^. IFN-γ-stimulated CTL and NK cell activity also results in a potent anti-tumor effect of IFN-γ-endostatin-based gene-radiotherapy in 4T1 tumor model^23^. Inhibition of B16 melanoma in mice by IFN-γ is synergized by IFN-α, while overexpressing interferon regulatory factor-2 results in resistance of B16 melanoma to endogenous IFN-γ^24^,^25^,^26^. Expression of IFN-γ by lung NK cells can resist the pulmonary metastasis. IFN-γ also functions as a co-stimulator with T cell costimulatory molecule B7 to activate the antitumor immune-response in mice^26^,^27^.

Taken together, although it needs more investigation to unveil the anti-tumorigenic properties of LXR and the involved mechanisms, in this study, we demonstrate that T0901317 at 5 mpk inhibits the development of lung tumors (both inoculated and carcinogen-induced carcinoma) which is correlated to activation of IFN-γ production. Our study also suggests that IFN-γ might be an important but not the sole molecular player in LXR-inhibited tumorigenesis.

## Methods

### Materials

LXR ligand (T0901317) was purchased from Cayman Chemical (Ann Arbor, MI). IFN-γ ELISA assay kit was purchased from eBioscience (San Diego, CA). Rabbit anti-IFN-γ, LXRα, LXRβ and Ki-67 polyclonal antibodies were purchased from Proteintech Group Inc. (Chicago, IL). Rabbit anti-TTF1 and SPC polyclonal antibodies were purchased from Santa Cruz Biotechnology, Inc. (Santa Cruz, CA). All other reagents were purchased from Sigma-Aldrich (St. Louis, MO) except where indicated.

### Animals

The protocols for *in vivo* study with mice were approved by the Ethics Committee of Nankai University (Tianjin, China), and the methods for *in vivo* study were carried out in accordance with the approved guidelines. BALB/c wild type mice, C57BL/6 wild type mice and IFN-γ^−/−^ mice were purchased from the Animal Center of Nanjing University (Nanjing, China) and maintained at the Animal Center of Nankai University with free access to water and food.

To study if LXR ligand can inhibit MCA/BHT-induced mouse lung adenocarcinomas or hyperplasia/inflammation, BALB/c or C57BL/6 wild type mice (~8 weeks old) were randomly divided into 3 groups (15 mice/group). All the animals were firstly i.p. injected with MCA solution in Mazolea oil (15 mg/kg bodyweight), and one week later, received weekly i.p. injection of BHT solution in Mazoea oil for next 6 weeks. The doses of BHT are: 1^st^ injection, 150 mg/kg bodyweight; 2^nd^ to 6^th^ injections, 200 mg/kg bodyweight. Mice in Group 1 were fed normal chow; mice in Group 2 were fed normal chow containing T0901317 [5 mg/day/kg bodyweight (mpk)] one week before MCA injection; and mice in Group 3 were fed normal chow containing T0901317 (5 mpk) from the last BHT injection (the same time of 6 weeks after MCA injection). To study if the inhibition of MCA/BHT-induced lung hyperplasia/inflammation by T0901317 is partially through induction of IFN-γ expression, IFN-γ^−/−^ mice (~8 weeks old) were randomly divided into 3 groups (12 mice/group) and received same treatment as C57BL/6 or BALB/c wild type mice as above described.

To study the effect of T0901317 on urethane-induced lung tumors, BALB/c mice (~8 weeks old) were randomly divided into 2 groups (15 mice/group). Mice were then fed normal chow (Group 1) and normal chow containing T0901317 (5 mpk, Group 2), respectively. After one week of T0901317 feeding, all the mice were i.p. injected with urethane (1 g/kg bodyweight) once every 3 days for 8 times. The experiment was terminated 60 days after the 1^st^ urethane injection.

To determine the effect of T0901317 on the growth of inoculated tumors, wild type mice were divided into 2 groups (15 mice/group) followed by feeding normal chow and normal chow containing T0901317 (5 mpk), respectively, for one week. All the mice were then injected with B16, EL4 or 4T1 cells (2 × 10^5^ cells/mouse, ~20 μl) with the following methods and continued T0901317 feeding: 1) B16 cells were subcutaneously (s.c.) injected in the right flank or orthotopically (o.t.) injected into the plantar region of the unilateral hind paws of mice^28^, respectively; 2) EL4 cells were also s.c. injected in the right flank; 3) 4T1 cells were o.t. injected at the nipple of female mouse. When the tumor masses were palpable, the longest dimension (length, L, mm) and shortest dimension (width, W, mm) were measured daily with a dial caliper. The tumor size (volume) was calculated as 0.5xLxW^2^ (mm^3^).

To determine if LXR ligand can prevent the metastasis of 4T1 tumors, after ~2 weeks of 4T1 cell injection, mice were i.p. injected with Luciferin solution (150 mpk, Caliper Life Sciences, Hopkinton, MA). About 10 min later, mice were fully anesthetized and scanned for the density and location of 4T1 tumors with IVIS spectrum (Xenogen, USA).

### Determination of tumor numbers, hyperplasia/inflammation areas, and expression of IFN-γ, Ki-67, SPC and TTF1 in the lung section by immnuohistochemical staining

At the end of experiment, mouse lung samples were collected and fixed with a 4% formalin solution followed by embedded in paraffin. The 5 μm step-sections collected from the paraffin blocks were stained with hematoxylin and eosin (HE) for histological evaluation with determination of tumor number and area with hyperplasia/inflammation, respectively.

Expression of IFN-γ, Ki-67, SPC and TTF1 in lung sections was determined by immunohistochemical staining. The sections were initially deparaffinised and hydrated. The antigen retrieval was obtained by heating the sections in a sodium citrate solution (0.01 M, pH 6.0) for 20 min in a 95 °C water bath. The sections were then blocked with goat serum for 15 min, followed by incubation with rabbit anti-IFN-γ, Ki-67, SPC or TTF1 polyclonal antibody overnight at 4 °C. After removal of primary antibody by washing with PBS, the sections were then incubated with biotin-conjugated goat anti-rabbit IgG for 15 min at RT. After washing with PBS, the sections were incubated in a horseradish peroxidase conjugated avidin solution for 20 min, followed by adding the developing solution. After development, the sections were stained with hematoxylin solution for the nucleus and then mounted under cover slides with permount. After adequate drying, the slides were observed and photographed with a microscope.

### Determination of IFN-γ, LXRα and LXRβ protein and mRNA expression in mouse lung by Western blot and real time RT-PCR

A piece of lung was removed at the end of experiment followed by extraction of cellular proteins. Expression of IFN-γ, LXRα and LXRβ protein in tissue protein extract was determined by Western blot as described^29^.

To determine mRNA expression, a piece of mouse lung was homogenized in Trizol reagent (Invitrogen, Grand Island, NY). The homogenate was well mixed with chloroform and spun for 10 min at 16,200 g at 4 °C. The top aqueous phase containing RNA was collected and mixed with an equal volume of isopropanol to precipitate total cellular RNA. About 1 μg of total cellular RNA was used to synthesize cDNA using a reverse transcription kit purchased from New England Biolabs (Ipswich, MA) followed by real time PCR with SYBR Green Master Mix (Bio-Rad, Los Angeles, CA) and the primers listed in Table 2. Expression of IFN-γ, LXRα or LXRβ mRNA was normalized by GAPDH mRNA in the corresponding samples.

### Determination of lipogenesis in IFN-γ^−/−^ mouse liver by Oil Red O staining and TG quantitative assay

To determine if LXR ligand can also induce lipogenesis in the liver of IFN-γ^−/−^ mice, IFN-γ^−/−^ mice were divided into two groups (5 mice/group), and fed normal chow and normal chow containing T0901317 (5 mpk), respectively, for two weeks followed by determination of lipid content in the liver with Oil Red O staining and triglyceride (TG) quantitative analysis. For Oil Red O staining, the liver frozen sections were prepared and soaked in PBS for 30 min and then stained with freshly prepared Oil Red O working solution (3 mg/ml in 60% isopropanol) for 40 min. After staining, the sections were rinsed with 60% isopropanol and lightly stained for nucleus with alum hematoxylin solution. The sections were finally rinsed with distilled water. Images of the sections were obtained with Leica DM3000 microscope (Wetzlar, Germany). To quantitatively analyze TG content in the liver, a piece of liver (~50 mg) was homogenized in 1.1 ml PBS. A portion of the homogenate (100 μl) was saved for determination of protein content which was used to normalize liver TG levels, and 1 ml of the homogenate was used to extract total lipids. TG level was determined as described^30^ using the assay kit which was purchased from Wako Chemicals.

### Data analysis

Data was generated from three independent experiments. All statistical results were obtained by Student’s t-test using Prism (GraphPad Software) except that the statistical analysis on survival curves was completed by log-rank test (SPSS Software). Significant difference was considered if P < 0.05.

## Additional Information

**How to cite this article**: Wang, Q. *et al.* Activation of liver X receptor inhibits the development of pulmonary carcinomas induced by 3-methylcholanthrene and butylated hydroxytoluene in BABL/c mice. *Sci. Rep.* **6**, 27295; doi: 10.1038/srep27295 (2016).

## Figures and Tables

**Table 1:** The death rate of MCA/BHT-injected BALB/c mice at the end of treatment.

	Total	Survived (w/tumor)	Survived (w/o tumor)	Died	Death rate
Group 1	15	5	4	6	40%
Group 2	15	4	8	3	20%*
Group 3	15	5	6	4	27%

BALB/c mice (15 mice/group) received treatment as scheduled in Fig. 2A. The number of mice survived with tumors (w/tumor) or survived without tumors (w/o tumor) or died was counted at the end of treatment. *P *<* 0.05 *vs.* Group 1 (n = 15).

**Table 2:** Sequences of the primers for real time RT-PCR analysis.

Gene	Forward	Backward
IFN-γ	GAGGAACTGGCAAAAGGATGGTGA	TGTTGTTGCTGATGGCCTGATTGT
LXRα	CCGGAATTCATGTCCTTGTGGCTGGAG	CGCGGATCCTCACTCGTGGACATCCC
LXRβ	TAGCCTCGAGCATGTCTTCCCCCACAAGTTC	CGACAAGCTTCTACTCGTGCACATCCCAGATC
GAPDH	ACAACTTTGGCATTGTGAA	GATGCAGGGATGATGTTCTG

IFN-γ, interferon-γ; LXRα, liver X receptor α; LXRβ, liver X receptor β; GAPDH, glyceraldehyde 3-phosphate dehydrogenase.
